# Insecticidal and repellent activities of pyrethroids to the three major pyrethroid-resistant malaria vectors in western Kenya

**DOI:** 10.1186/1756-3305-7-208

**Published:** 2014-05-02

**Authors:** Hitoshi Kawada, Kazunori Ohashi, Gabriel O Dida, George Sonye, Sammy M Njenga, Charles Mwandawiro, Noboru Minakawa

**Affiliations:** 1 Department of Vector Ecology & Environment, Institute of Tropical Medicine, Nagasaki University, Nagasaki, Japan; 2 Health and Crop Sciences Research Laboratory, Sumitomo Chemical Co Ltd, Hyogo, Japan; 3 School of Public Health, Maseno University, Kisumu, Kenya; 4 Springs of Hope, Mbita, Kenya; 5 Eastern and Southern Africa Centre of International Parasite Control, Nairobi, Kenya; 6 Kenya Medical Research Institute, Nairobi, Kenya; 7 The Global Center of Excellence Program, Nagasaki University, Nagasaki, Japan

**Keywords:** Permethrin, Deltamethrin, Resistance, Repellency, *Anopheles gambiae* s.s, *Anopheles arabiensis*, *Anopheles funestus*, LLIN, Kenya

## Abstract

**Background:**

The dramatic success of insecticide treated nets (ITNs) and long-lasting insecticidal nets (LLINs) in African countries has been countered by the rapid development of pyrethroid resistance in vector mosquitoes over the past decade. One advantage of the use of pyrethroids in ITNs is their excito-repellency. Use of the excito-repellency of pyrethroids might be biorational, since such repellency will not induce or delay the development of any physiological resistance. However, little is known about the relationship between the mode of insecticide resistance and excito-repellency in pyrethroid-resistant mosquitoes.

**Methods:**

Differences in the reactions of 3 major malaria vectors in western Kenya to pyrethroids were compared in laboratory tests. Adult susceptibility tests were performed using World Health Organization (WHO) test tube kits for F1 progenies of field-collected *An. gambiae* s.s., *An. arabiensis*, and *An. funestus* s.s., and laboratory colonies of *An. gambiae* s.s. and *An. arabiensis*. The contact repellency to pyrethroids or permethrin-impregnated LLINs (Olyset® Nets) was evaluated with a simple choice test modified by WHO test tubes and with the test modified by the WHO cone bioassay test.

**Results:**

Field-collected *An. gambiae* s.s., *An. arabiensis*, and *An. funestus* s.s. showed high resistance to both permethrin and deltamethrin. The allelic frequency of the point mutation in the voltage-gated sodium channel (L1014S) in *An. gambiae* s.s. was 99.3–100%, while no point mutations were detected in the other 2 species. The frequency of takeoffs from the pyrethroid-treated surface and the flying times without contacting the surface increased significantly in pyrethroid-susceptible *An. gambiae* s.s. and *An. arabiensis* colonies and wild *An. arabiensis* and *An. funestus* s.s. colonies, while there was no significant increase in the frequency of takeoffs or flying time in the *An. gambiae* s.s. wild colony.

**Conclusion:**

A different repellent reaction was observed in the field-collected *An. gambiae* s.s. than in *An. arabiensis* and *An. funestus* s.s. It might be that resistant mosquitoes governed by knockdown resistance (*kdr*) loose repellency to pyrethroids, whereas those lacking *kdr* maintain high repellency irrespective of their possessing metabolic resistance factors to pyrethroids. Further genetic evaluation is required for the demonstration of the above hypothesis.

## Background

Pyrethroids are the predominant insecticides and are used in various formulations for mosquito control. Globally, pyrethroids comprise 40% of the insecticides used annually for indoor residual spraying against malaria vectors [1]. Since the World Health Organization (WHO) adopted the use of long-lasting insecticidal nets (LLINs) as a principal strategy for effective malaria control in the Roll Back Malaria Partnership [2], pyrethroids have been the only class of insecticides used for LLINs [1,3]. Pyrethroids have unique modes of action such as fast knockdown and excito-repellent effects [4].

A dramatic success of insecticide treated nets (ITNs) and LLINs was recorded in African countries. However, it has been countered by the rapid development of pyrethroid resistance in vector mosquitoes over the past decade [5]. Pyrethroid resistance has developed in the African malaria vectors primarily through 2 resistance mechanisms. The first is resistance at the target site, in which only 1 point mutation at 1014 L (L1014F or L1014S) in the voltage-gated sodium channel causes insensitivity to pyrethroids, resulting in knockdown resistance (*kdr*). The second is metabolic resistance that relates to the elevated activity of 1 or more detoxification enzymes (cytochrome P450s, etc.). The distribution patterns of the types of point mutations in voltage-gated sodium channels in African malaria vectors are unique [5-7]. The point mutations in the voltage-gated sodium channel are commonly reported in *Anopheles gambiae* Giles (*An. gambiae* sensu stricto [s.s.]). L1014F mutations are widespread in western African countries, L1014S mutations are distributed in eastern Africa, and L1014F/L1014S hybrids in *An. gambiae* s.s. are reported in the central region. In contrast, such *kdr* mutations do not seem to be common in *An. arabiensis* Patton [8-10], but the metabolic resistance seems to be most common in this species [10,11]. Fortunately, *kdr* mutations have not been reported in African malaria vectors except for these 2 species. Some metabolic resistance, however, has been reported in the *An. funestus* Giles group [11-14]. Additionally, the co-occurrence of these 2 resistance factors (metabolic and *kdr*) in a single mosquito population would lead to further threats to malaria control, since both factors might reinforce the resistance of each other.

Darriet et al. reported that, even in areas where *An. gambiae* s.s. was resistant to permethrin and deltamethrin (>90% *kdr*), bed nets treated with these insecticides remained effective [15]. This apparent paradox was explained by behavioral changes in the resistant mosquitoes; they were less repelled by the insecticide, remained on the pyrethroid-treated material for longer periods, and thus received a higher dose of insecticide [16]. On the other hand, excito-repellency is thought to be an advantage of the use of pyrethroids, which provide personal protection from mosquito bites even when ITNs accumulate holes during the course of daily usage [17]. Use of the excito-repellency of pyrethroids might be biorational, since such repellency will not induce or delay the development of any physiological resistance since it does not kill the affected insects or reduce the chance of contact to the insecticides and causes low selection pressure on insect populations [18,19]. However, little is known about the relationship between the mode of insecticide resistance and excito-repellency in pyrethroid-resistant mosquitoes.

The aim of this study was to investigate the difference in the repellent reaction of pyrethroid resistant vector mosquitoes to pyrethroids and pyrethroid-impregnated nets.

## Methods

### Mosquito collection site

The mosquito collection sites were located in the Mbita and Suba districts of Nyanza Province in western Kenya. The rainfall pattern in the area is bimodal, with a long rainy season occurring from March to May and a short rainy season occurring in November and December. Malaria infection rates rise steadily between September and February and peak briefly in June, following the long rains [20]. The Mbita and Suba districts have been identified as high vector transmission areas in Kenya, and more than 50% of the population is exposed to malaria at a rate of ≥40% *Pf*PR_2–0_ (*Plasmodium falciparum* parasite rate corrected to a standard age range of 2 to <10 y) [21].

### Insecticide susceptibility tests using WHO test tubes

**Figure 1:**
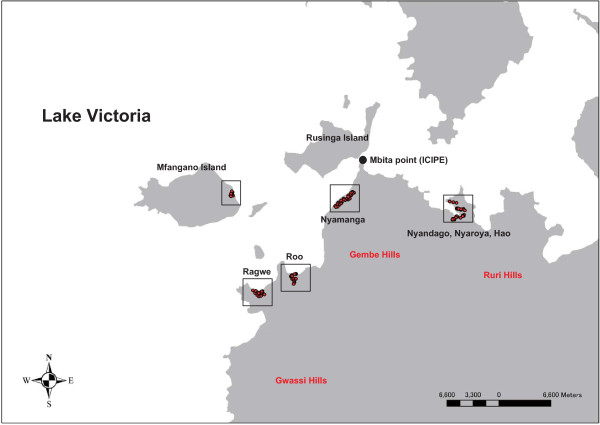
**Map of the sites for mosquito collection.** Red circles indicate houses used for mosquito collection.

Indoor collection of adult mosquitoes was performed in houses in Nyandago, Nyaroya, Hao (E34°18′–E34°19′, S0°27′–S0°28′), and Nyamanga villages (E34°10′–E34°12′, S0°26′–S0°28′) in the Gembe area in Mbita District on the eastern side of Lake Victoria; Roo, Ragwe villages (E34°04′–E34°08′, S0°32′–S0°35′) in Suba District on the western side of Lake Victoria; and Mfangano Island (E34°03′–E34°04′, S0°27′–S0°28′) (Figure 1). Collections were performed from May 11th to July 6th, 2011, using a battery-powered aspirator (C-Cell Aspirator; BioQuip Products, CA, USA) between 7:00 and 9:00 AM by 3 people. After collection, blood-fed and gravid female mosquitoes were individually confined in a 20-mL glass vial containing ca. 2 mL of dechlorinated tap water. A strip of filter paper (approximately 3 × 4 cm) was placed inside each vial to collect eggs. F1 larvae from the separate egg batches were pooled into 1 batch of the same species after identification with PCR and reared with dechlorinated tap water until adult emergence. Larvae were fed a 1:1 mixture of powdered animal food (CE-2; Clea Inc., Tokyo, Japan) and dried yeast (Ebios®; Mitsubishi Tanabe Pharma, Tokyo, Japan).

Adult susceptibility tests with insecticides were performed using WHO test tube kits for F1 progenies of field-collected *An. gambiae* s.s., *An. arabiensis*, *An. funestus* s.s., and the laboratory colonies of *An. gambiae* s.s. and *An. arabiensis* reared at the International Center of Insect Physiology and Ecology (ICIPE, Mbita, Nyanza, Kenya) according to WHO instructions (WHO/CDS/CPC/MAL/98.12). Papers impregnated with 0.75% permethrin, 0.05% deltamethrin, 0.1% propoxur, or 1.0% fenitrothion were used for the tests. Ten 1- to 3-day-old unfed female mosquitoes were released into WHO test tubes for exposure to an insecticide-impregnated paper for 1 hour, and the time to knockdown was recorded. Insects were then transferred to a clean tube and fed via cotton soaked with a 5% glucose solution, and mortality was recorded after 1 day. The time required for 50% knockdown (KT_50_) was obtained, and average mortality was calculated. Two to 4 replications were performed for each insecticide.

### Contact repellency test using WHO test tubes

The contact repellency of F1 progenies of field-collected *An. gambiae* s.s., *An. arabiensis*, and *An. funestus* s.s. was evaluated with a simple choice test modified by Grieco et al. [22] using WHO test tubes. Papers impregnated with 0.75% permethrin and 0.05% deltamethrin were used. Ten 1- to 3-day-old female mosquitoes were transferred into a WHO test tube lined with untreated paper that was connected to another test tube lined with an insecticide paper. Just after releasing the shutter, mosquitoes were transferred into a tube with an insecticide paper by blowing. The test tube was placed horizontally in a dark container to avoid the influence of light for 10 min with a shutter kept open. The shutter was closed after 10 min, and the number of mosquitoes in the tube with untreated paper (number repelled) was counted. For every test a control assay was performed in which an untreated paper was used in place of the insecticide-treated paper. Three to 7 replicates were performed with each insecticide.

### Contact repellency test modified by the WHO cone bioassay test

The contact repellency of adult mosquitoes was evaluated by the test modified by the WHO cone bioassay test (WHO/CDS/WHOPES/GCDPP/2005.11). F1 progenies of field-collected *An. gambiae* s.s., *An. arabiensis*, and *An. funestus* s.s., and the laboratory colonies of *An. gambiae* s.s. and *An. arabiensis* reared at the ICIPE were used for the test. Each 1- to 3-day-old female mosquito was exposed to Olyset® Net materials (25 × 25 cm) or a paper impregnated with 0.75% permethrin for a WHO test tube assay for 3 minutes under standard WHO cones. A control assay using an insecticide-untreated filter paper was performed concurrently using another female mosquito of the same colony. After releasing a mosquito, the number of takeoffs from the net surface and the cumulative flying time after taking off to the next touchdown over 3 minutes was recorded. A total of 21, 22, and 5 replicates were performed for *An. gambiae* s.s., *An. arabiensis*, and *An. funestus* s.s., respectively. Because of the short time contact (3 minutes), neither mortality nor knockdown was observed during the test.

### Species identification

Adult mosquitoes were examined microscopically to distinguish *An. gambiae* s.l. and *An. funestus* s.l. from other anophelines based on identification keys developed by Gillies and Coetzee [23]. Multiplex PCR methods described by Scott et al. [24] and Koekemoer et al. [25] were used for species identification.

### Detection of point mutations in the voltage-gated sodium channel

PCR and direct DNA sequencing were used to identify point mutations at 1014 L in the field-collected mosquitoes according to the method of Kawada et al. [10,11]. The legs of an adult were homogenized in a mixed solution of extraction solution (20 μl) + tissue preparation solution (5 μl) (REDExtract-N-Amp™ Tissue PCR Kit; SIGMA, St. Louis, MO, USA) for extraction of DNA. Initial fragment amplification was carried out using primers AGKF1 (CATGATCTGCCAAGATGGAA) and AGKR1 (GTTGGTGCAGACAAGGATGA) for *An. gambiae* s.l.; and AFF1 (ACCAAGATCTGCCAAGATGG) and AFR1 (TGGTGCAGACAAGGATGAAG) for *An. funestus* s.s., respectively. The PCR mixture contained 4 μl of REDExtract-N-Amp™ ReadyMix (SIGMA), 0.5 μM of each primer, and 1 μl of the DNA template in a total volume of 10 μl. PCR was performed under the following conditions: 94°C for 3 min and 35 cycles of 94°C for 15 s, 55°C for 30 s, 72 °C for 30 s, and 72°C for 10 min (for *An. gambiae* s.l.) or 94°C for 3 min and 35 cycles of 94°C for 15 s, 45°C for 30 s, 72°C for 30 s, and 72°C for 10 min (for *An. funestus* s.s.). DNA sequencing was carried out using primers Dg1 (TGGATHGARWSHATGTGGGAYTG) for *An. gambiae* s.l. and Dg3 (TGGATCGAATCCATGTGGGACTG) for *An. funestus* s.s., respectively. A BigDye Terminator v. 3.1 Cycle Sequencing Kit (Applied Biosystems Japan Ltd., Tokyo, Japan) was used for DNA sequencing according to the manufacturer’s instructions. Direct DNA sequencing was performed using the 3730 DNA Analyzer (Applied Biosystems). The electropherogram of the targeted amino acid replacement was analyzed using MEGA 4.0 public domain software (http://www.megasoftware.net/). The unique DNA haplotype sequences were deposited into GenBank.

### Data analysis

A digital map in shape file format (Kenya-Boundaries, FAO Africover, http://www.africover.org/index.htm) was used for mapping the collection sites. The geographical positions of the collection sites were plotted on the map using ArcGIS 10.1 (ESRI Japan Corp, Tokyo, Japan).

The median knockdown times (KT_50_s) in the insect susceptibility test were calculated using the Bliss’ probit method [26]. The mosquito repellency in the simplified contact repellency test using WHO test tubes was calculated as the mean percentage of female mosquitoes repelled into untreated tubes corrected by a control test. The square root of the percent of repellency in each test was arcsin converted, and analysis of variance (ANOVA) and the multiple comparison of the repellency by a Tukey honestly significant difference test were performed using R × 64 Ver. 2.15.1 (http://www.R-project.org). The comparison of the repellency of the Olyset® Net, permethrin-impregnated paper, and untreated paper in the contact repellency test modified by the WHO cone test was performed with a Kruskal-Wallis test using JMP 7.0 J (SAS Institute Japan Inc., Tokyo, Japan).

### Ethics statement

The protocol for the study (case no. 1775) was approved by the Scientific Steering Committee and the National Ethics Review Committee of the Kenya Medical Research Institute. All necessary permits were obtained for the described field studies. No mosquito collection was done without the approval of the head of the village and the owner and occupants of the collection house.

## Results

### Insecticidal susceptibility of F1 female adults of field-collected *An. gambiae* s.s., *An. arabiensis*, and *An. funestus* s.s

**Figure 2:**
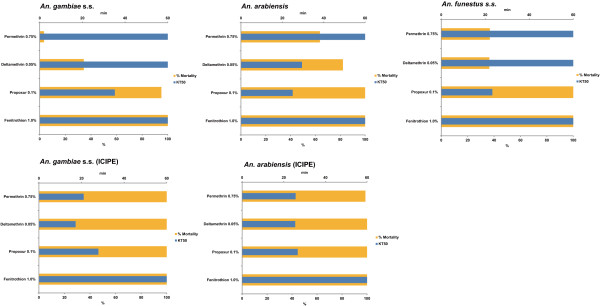
**Mortalities and KT**_**50**_**s of the laboratory colonies (ICIPE) and F1 progenies of field-collected *An. gambiae *** **s.s., *An. arabiensis*, and *An. funestus *** **s.s. using WHO test tubes.**

No mortality was observed in control groups in the susceptibility tests. F1 adults of the field-collected *An. gambiae* s.s., *An. arabiensis*, and *An. funestus* s.s. showed high resistance to both permethrin (3.4%, 63.6%, and 36.8% mortality, respectively) and deltamethrin (34.4%, 82.1%, and 36.4% mortality, respectively). The KT_50_s of the 3 species for permethrin and deltamethrin were >60 minutes except for deltamethrin in *An. arabiensis* (KT_50_, 29.5 minutes), indicating low knockdown activities of the 2 pyrethroids against these mosquitoes as well as low killing activities. In contrast, lethal activities against *An. gambiae* s.s., *An. arabiensis*, and *An. funestus* s.s. of fenitrothion (100% mortality for 3 species) and propoxur (100%, 100%, and 95.0% mortality, respectively) were higher than those of the 2 pyrethroids. The knockdown activity of propoxur (KT_50_, 23.2, 25.0, and 35.2 minutes, respectively) was higher than that of the 2 pyrethroids. The laboratory colonies of *An. gambiae* s.s. and *An. arabiensis* reared in ICPE showed high susceptibility to all of the insecticides used (Figure 2). The allelic frequency of the point mutation in the voltage-gated sodium channel (L1014S) in F1 *An. gambiae* s.s. was 100% in Ragwe (n = 214, Accession AB776709) and 99.3% in Mfangano (n = 211, Accession AB776707, AB776708), while not a single point mutation was detected in the other 2 species, indicating that the major pyrethroid resistance mechanism in this species was *kdr*.

### Contact repellency of pyrethroids against F1 female adults of field-collected mosquitoes

**Figure 3:**
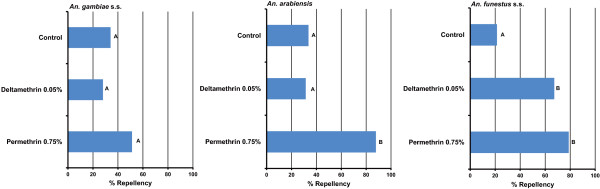
**Contact repellency of permethrin and deltamethrin against the F1 progenies of *An. gambiae *s.s., *An. arabiensis*, and *An. funestus *s.s. collected in the study area by a simplified contact repellency test modified by Grieco et al. [**[22]**] using WHO test tubes.** Repellency was calculated as the average percentage of mosquitoes in the untreated test tubes. Different alphabetical letters indicate significant differences by means of a Tukey honestly significant difference test.

The contact repellency of permethrin 0.75% paper and deltamethrin 0.05% paper in the simplified contact repellency test using WHO test tubes is shown in Figure 3. The repellency (the average percentage of mosquitoes in the untreated test tubes) in the control test in *An. gambiae* s.s., *An. arabiensis*, and *An. funestus* s.s. was 34.1%, 33.6%, and 21.4%, respectively. Significant differences in repellency were observed in *An. arabiensis* (ANOVA, df = 2, F = 15.68, p = 0.00014) and *An. funestus* s.s. (ANOVA, df = 2, F = 23.15, p = 0.00047), but not in *An. gambiae* s.s. (ANOVA, df = 2, F = 1.99, p = 0.166), among the 3 test regimens. The repellency of permethrin 0.75% paper was significantly higher in *An. arabiensis* (87.9%, p < 0.001) and *An. funestus* s.s. (78.8%, p < 0.001) compared with the control test. In contrast, the repellency of deltamethrin 0.05% paper was significant in *An. funestus* s.s. (67.3%, p = 0.0023) but not in *An. arabiensis* (31.4%, p = 0.66).

**Figure 4:**
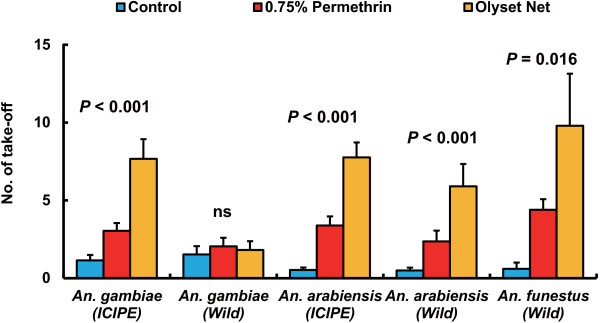
**Number of takeoffs of female *An. gambiae *s.s., *An. arabiensis*, and *An. funestus *s.s. during a 3-min exposure to an Olyset® Net, 0.75% permethrin paper, or untreated net material by modified WHO cone bioassay.** Figures indicate the significance levels by a Kruskal-Wallis test. Bars indicate SEs.

**Figure 5:**
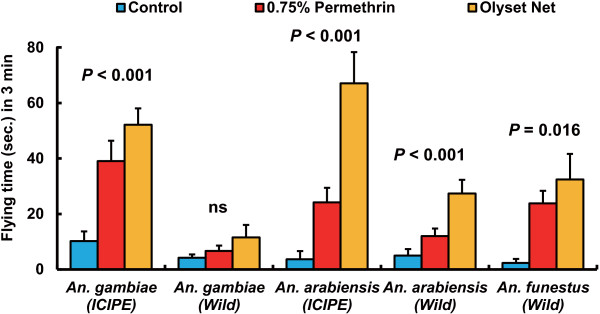
**Cumulative flying time of female *An. gambiae *s.s., *An. arabiensis*, and *An. funestus *s.s. during a 3-min exposure to an Olyset® Net, 0.75% permethrin paper, or untreated net material by modified WHO cone bioassay.** Figures indicate the significance levels by a Kruskal-Wallis test. Bars indicate SEs.

The contact repellency of permethrin 0.75% paper and an Olyset® Net against laboratory colonies (ICIPE colonies) and field-collected F1 colonies (wild colonies) of *An. gambiae* s.s. and *An. arabiensis*, and a wild colony of *An. funestus* s.s. by a modified WHO cone assay is shown in Figures 4 and 5. The number of takeoffs in the control test was 1.1 ± 0.3 (ICIPE colony) and 1.5 ± 0.5 (wild colony) in *An. gambiae* s.s., 0.5 ± 0.2 (ICIPE colony) and 0.5 ± 0.2 (wild colony) in *An. arabiensis*, and 0.6 ± 0.4 (wild colony) in *An. funestus* s.s. Significant differences in the number of takeoffs were observed in the *An. gambiae* s.s. ICIPE colony (Kruskal-Wallis test, df = 2, *χ*^2^ = 24.0, p < 0.0001), *An. arabiensis* ICIPE colony (Kruskal-Wallis test, df = 2, *χ*^2^ = 38.3, p < 0.0001), *An. arabiensis* wild colony (Kruskal-Wallis test, df = 2, *χ*^2^ = 20.6, p < 0.0001), and *An. funestus* s.s. wild colony (Kruskal-Wallis test, df = 2, *χ*^2^ = 8.3, p = 0.016), indicating that the number of takeoffs in these colonies significantly increased after exposure to permethrin 0.75% paper and an Olyset® Net. In contrast, there was no significant difference in the number of takeoffs in the *An. gambiae* s.s. wild colony (Kruskal-Wallis test, df = 2, *χ*^2^ = 0.70, p = 0.698) (Figure 4).

Total flying times (in seconds) over 3 minutes in the control test were 10.2 ± 3.5 (ICIPE colony) and 4.2 ± 1.2 (wild colony) in *An. gambiae* s.s., 3.6 ± 3.0 (ICIPE colony) and 5.0 ± 2.3 (wild colony) in *An. arabiensis*, and 2.3 ± 1.4 (wild colony) in *An. funestus* s.s. Significant differences in total flying times were observed in the *An. gambiae* s.s. ICIPE colony (Kruskal-Wallis test, df = 2, *χ*^2^ = 21.4, p < 0.0001), *An. arabiensis* ICIPE colony (Kruskal-Wallis test, df = 2, *χ*^2^ = 35.2, p < 0.0001), *An. arabiensis* wild colony (Kruskal-Wallis test, df = 2, *χ*^2^ = 16.5, p = 0.0003), and *An. funestus* s.s. wild colony (Kruskal-Wallis test, df = 2, *χ*^2^ = 8.2, p = 0.016), indicating that the total flying time in these colonies also significantly increased after exposure to permethrin 0.75% paper and an Olyset® Net. In contrast, there was no significant difference in the total flying time in the *An. gambiae* s.s. wild colony (Kruskal-Wallis test, df = 2, *χ*^2^ = 0.50, p = 0.775) (Figure 5).

## Discussion

High pyrethroid resistance in 3 major malaria vectors was observed in the study area as previously reported [11,14]. Kawada et al. [11] reported that the resistance mechanisms were multimodal, including *kdr*, caused by the point mutation of the voltage-gated sodium channel (L1014S) in *An. gambiae* s.s., and mixed cytochrome P450-related metabolic factors in both *An. arabiensis* and *An. funestus* s.s. In Nyanza province, dieldrin was reported to be administered mainly through aerial spraying, especially for tsetse fly control [27], while the organized intensive spraying of DDT for mosquito control was not performed in the 1970s and 1980s, and no IRS has been administered since then. Therefore, the extensive use of ITNs and LLINs in the study area is thought to be a major factor causing such high pyrethroid resistance. Fortunately, the present results showing no cross-resistance in carbamate- and organophosphate-class insecticides might provide a specific remedy for the emergence control of the vectors in this area. Two ICIPE laboratory colonies of *An. gambiae* s.s. and *An. arabiensis* were collected in the same field as in this study (ca. 10 years ago in *An. gambiae* s.s. and several years ago in *An. arabiensis*). The frequency of allelic L1014S point mutations in the laboratory colony of *An. gambiae* s.s. was 6.3% [11], while that in field-collected *An. gambiae* s.s. was >99%. The historical increase in *kdr* mutations in *An. gambiae* s.s. after the spread in the use of ITNs and LLINs in 2000s, as reported by Mathias et al. [6], has also been found in our study area (unpublished data), indicating that the *An. gambiae* s.s. ICIPE colony has been maintaining high susceptibility to pyrethroids with their low frequency *kdr* mutations. On the other hand, the cytochrome P450-related resistant factors [11] in the laboratory colony of *An. arabiensis* were thought to be absent when they were transferred into the laboratory from the field, or otherwise to have declined in the course of generations of rearing. Such a decline in resistance without selection pressure from insecticides in the latter case might sometimes be common in the laboratory colonies with metabolic resistance factors [28].

The repellency of permethrin (0.75% paper) was significantly higher than that of 1/15 the amount of deltamethrin (0.05%) in the wild colony of *An. arabiensis*. Siegert et al. [29] pointed out the same difference in repellency between an Olyset® Net (containing 1000 mg of permethrin per square meter) and a PermaNet® (55 mg of deltamethrin per square meter). The authors reported that the Olyset® Net reduced the landing attempts of mosquitoes and elevated their flight frequency, while there was a higher incidence of mosquito landing attempts on the PermaNet® [28]. The difference in repellency between the 2 pyrethroids might be explainable by their structural differences (permethrin belongs to the non-cyano-containing Type I pyrethroids and deltamethrin to the alpha-cyano-containing Type II pyrethroids) resulting in the different neurotoxicity [30].

It is interesting that the lack of repellency to pyrethroids was observed only in the wild colony of *An. gambiae* s.s. even though the 2 other wild colonies also possess high resistance to pyrethroids. It might be reasonable to consider that the point mutation in the voltage-gated sodium channel interferes with the sensitivity of the sensory nervous system to pyrethroids as well as with the central nervous system, causing less irritancy to mosquitoes, resulting in slower avoidance or reduced repellency [18]. However, the phenomena previously reported on the relationships between *kdr* and pyrethroid repellency were not simple. Chandre et al. [31] reported that a laboratory-selected *An. gambiae* s.s. colony originating from Burkina Faso with a homozygous *kdr* factor (RR) lost contact repellency to permethrin 1% impregnated paper as compared to an insecticide susceptible Kisumu colony (SS) and a *kdr* heterozygous colony (RS). RS hybrids in the above report were exactly intermediate between RR and SS individuals in repellency. Corbel et al. [32] also reported a non-linear relationship in the survivorships of RR, RS, and SS genotypes with permethrin dosage, and higher dosages of permethrin more efficiently killed the RS genotypes of *An. gambiae* s.s. than did lower dosages. The authors concluded that heterozygous mosquitoes (RS) were more efficiently killed than susceptible mosquitoes (SS), since *kdr* resistance to the irritant effect appeared to be co-dominant while resistance to the lethal effect was recessive [31], so the RS mosquitoes stayed longer than SS mosquitoes on the permethrin-treated surface, picking up more insecticide and being killed in higher proportions. There was no answer, however, to the question of why the irritant effect of *kdr* was co-dominant while the lethal effect of the same gene was recessive. The *kdr*-governed absence of pyrethroid repellency was also reported by Virgona et al. [33] in houseflies. The authors suggested that both *kdr* and *pen* factors play a significant role in the repellency resistance of houseflies, since the *pen* gene causes a reduced rate of penetration of insecticides [34] and possibly also reduces the amount of pyrethroids reaching the sensory nerves, which are closely related to repellency. Although the relationships or synergisms between *kdr* and *pen* have not been well clarified, it seems to be more plausible to assume the existence of the second factors, which synergize the repellency caused by *kdr* gene.

Chandre et al. [31] and Corbel et al. [32] concluded that a high proportion of *An. gambiae* s.s. possessing homozygous *kdr* mutations were killed by prolonged contact with pyrethroids because of their reduced sensitivity to the excito-repellent effects of pyrethroids, and that permethrin-treated nets seemed unlikely to select for pyrethroid resistance in areas where the *kdr* mutation is rare and present mainly in heterozygous form. The above hypothesis seems to be incorrect since the *kdr* mutations in *An. gambiae* s.s. populations seem to have increased in accordance with the extensive use of LLINs from a low allelic percentage to the maximum (>90%) [6]. Recently, Kawada et al. found that permethrin-impregnated LLIN were effective against the 3 major pyrethroid-resistant malaria vectors, *An. gambiae* s.s., *An. arabiensis*, and *An. funestus* s.s., in western Kenya, since the frequency of human feeding was found to be reduced to a low level during ‘bedtime’. However, the large proportion of human blood feeding was shown to take place during the time people were active outside LLINs in both *An. arabiensis* and *An. funestus* s.s., whereas no such event was shown in *An. gambiae* s.s. [unpublished data]. One plausible explanation for such a species-dependent difference in host feeding activities under LLIN use might be the difference in the repellency to pyrethroids observed in the present report. The historical population decline of *An. gambiae* s.s. reported by Bayoh et al. [35] might also be explained by the above behavioral characteristics in the *An. gambiae* s.s. population. *Anopheles gambiae* s.s. might have not changed its characteristics as a “midnight feeder” because of the lack of repellency despite the existence of LLINs, and it still has to rely on the limited human blood sources, most of which are protected by LLINs, resulting in the decline in their population size.

## Conclusion

A different repellent reaction was observed in the field-collected *An. gambiae* s.s. than in *An. arabiensis* and *An. funestus* s.s. It might be that resistant mosquitoes governed by knockdown resistance (*kdr*) loose repellency to pyrethroids, whereas those lacking *kdr* maintain high repellency irrespective of their possessing metabolic resistance factors to pyrethroids. The comparative studies of the repellency of mosquitoes have mainly studied *kdr*, and those studying enhanced metabolic factors have been unexpectedly few. Further genetic evaluation should be required to demonstrate the above hypothesis.

## Competing interests

The authors declare that they have no competing interests.

## Authors’ contributions

HK and KO designed the study, carried out the experiments, and drafted the manuscript. GOD and GS arranged the field studies, obtained informed consents from the participants for the study, and organized the staff for the experiments. SMN, CM, and NM critically revised the protocol for the study. All authors read and approved the final version of the manuscript.
